# Xeroderma Pigmentosum C (XPC) Mutations in Primary Fibroblasts Impair Base Excision Repair Pathway and Increase Oxidative DNA Damage

**DOI:** 10.3389/fgene.2020.561687

**Published:** 2020-11-27

**Authors:** Nour Fayyad, Farah Kobaisi, David Beal, Walid Mahfouf, Cécile Ged, Fanny Morice-Picard, Mohammad Fayyad-Kazan, Hussein Fayyad-Kazan, Bassam Badran, Hamid R. Rezvani, Walid Rachidi

**Affiliations:** ^1^University Grenoble Alpes, SyMMES/CIBEST UMR 5819 UGA-CNRS-CEA, Grenoble, France; ^2^Laboratory of Cancer Biology and Molecular Immunology, Faculty of Sciences I, Lebanese University, Hadath, Lebanon; ^3^University Grenoble Alpes, CEA, Inserm, BIG-BGE U1038, Grenoble, France; ^4^Université de Bordeaux, Inserm, BMGIC, U1035, Bordeaux, France; ^5^Centre de Référence pour les Maladies Rares de la Peau, CHU de Bordeaux, Bordeaux, France

**Keywords:** Xeroderma Pigmentosum C, nucleotide excision repair, base excision repair, ultra violet (UV) light, oxidative DNA damage, oxidative stress, skin cancer

## Abstract

Xeroderma Pigmentosum C (XPC) is a multi-functional protein that is involved not only in the repair of bulky lesions, post-irradiation, via nucleotide excision repair (NER) *per se* but also in oxidative DNA damage mending. Since base excision repair (BER) is the primary regulator of oxidative DNA damage, we characterized, post-Ultraviolet B-rays (UVB)-irradiation, the detailed effect of three different XPC mutations in primary fibroblasts derived from XP-C patients on mRNA, protein expression and activity of different BER factors. We found that XP-C fibroblasts are characterized by downregulated expression of different BER factors including *OGG1*, *MYH*, *APE1*, *LIG3*, *XRCC1*, and *Pol*β. Such a downregulation was also observed at OGG1, MYH, and APE1 protein levels. This was accompanied with an increase in DNA oxidative lesions, as evidenced by 8-oxoguanine levels, immediately post-UVB-irradiation. Unlike in normal control cells, these oxidative lesions persisted over time in XP-C cells having lower excision repair capacities. Taken together, our results indicated that an impaired BER pathway in XP-C fibroblasts leads to longer persistence and delayed repair of oxidative DNA damage. This might explain the diverse clinical phenotypes in XP-C patients suffering from cancer in both photo-protected and photo-exposed areas. Therapeutic strategies based on reinforcement of BER pathway might therefore represent an innovative path for limiting the drawbacks of NER-based diseases, as in XP-C case.

## Introduction

Skin is considered a first line of defense protecting the human body against several chemical and physical stressors (such as microbial infections, irradiation, toxic substances, pollutants) that can generate molecular DNA lesions at a rate of 1,000 to 1,000,000 lesions per cell per day (Kelley, 2012). Such lesions could be repaired via different repair systems [such as base excision repair (BER), nucleotide excision repair (NER), mismatch repair (MMR)] that are specialized to remove DNA damage and maintain genome integrity. Ultraviolet B rays (UVB) (280–315 nm), an environmental stress, could act as a carcinogen that triggers tumor-initiation, -promotion, and progression. UVB, by inducing both direct and indirect DNA damage, is capable of causing genomic instability, thus leading to acute- or delayed-skin lesions (D’Orazio et al., 2013; Melis et al., 2013a). Direct lesions, including pyrimidine (6–4) pyrimidone photoproducts [(6–4) PPs] and cyclobutane pyrimidine dimers (CPDs), can cause UV-signature mutations (C > T and CC > TT transition mutations). Such mutations usually contribute to a dominant phenotype as in the case of *p53* gene mutations that are dominant in skin cancer (Ravanat et al., 2001; Kemp et al., 2017). Indirect damages, such as 8-oxoguanine (8-oxoGua), are oxidative DNA damage occurring at a rate of 10^4^ hits per cell per day in humans and are usually triggered by UV-induced reactive oxygen species (ROS) that will also damage protein and lipid cellular molecules. However, unlike these molecules, DNA lesions are not replaced with new molecules rather repaired (Ravanat et al., 2001; Rezvani et al., 2006; Melis et al., 2013b). If left unrepaired, 8-oxoGua may give rise to the oxidative stress hallmark, GC→TA transversion mutation, subsequently, sporadic and hereditary cancerogenesis (Hegde et al., 2008). Therefore, these oxidized bases are repaired via BER pathway for maintaining genome integrity and survival, consequently preventing cancer and aging (David et al., 2007; Hegde et al., 2008; Krokan and Bjørås, 2013). In general, BER corrects small base lesions from oxidation, deamination and alkylation in the nucleus and mitochondria. First, depending on the type of lesions and cell’s physiological state, a selective DNA glycosylase will recognize and remove the base lesion, leaving an abasic site that is further processed by short-patch or long-patch repair. The subsequent steps are incision, end-processing, repair synthesis, and ligation (Krokan and Bjørås, 2013). Meanwhile, direct bulky photoproducts (CPDs and (6–4) PPs) are removed by NER pathway to prevent UV-mediated mutagenesis and maintain cell and tissue viability post-stress. NER is regulated by DNA damage-induced signaling pathway (DDR pathway) and subdivided into global genome NER (GG-NER) and RNA-polymerase dependent transcriptional coupled NER (TC-NER) (Park and Kang, 2016; Kemp et al., 2017). Both sub-pathways differ in their recognition step, speed, and efficiency. Hereditary alterations in NER-genes may result in severe diseases, such as Cockayne syndrome, Trichothiodystrophy, and Xeroderma Pigmentosum (XP) (Park and Kang, 2016).

Xeroderma Pigmentosum is a rare, recessive, cancer-prone, autosomal genodermatosis with an incidence rate of 1 in 250,000 in North America, and 1 in 1,000,000 in Europe (Lehmann et al., 2011; Pázmándi et al., 2019). Its prevalent symptoms include photosensitivity, cutaneous atrophy, dry pigmented-freckled skin, and a 2,000 and 10,000-fold incidence increase of melanoma and non-melanoma skin cancers, respectively. It is characterized by the accumulation of mutations either in proto-oncogenes (such as *BRAF* and *MYC*) or tumor suppressor genes (such as *P53* and *PTCH1*) that persist due to NER defect whereby neither DNA repair nor apoptosis occurs (Daya-Grosjean and Sarasin, 2004; Murray et al., 2016; Zebian et al., 2019). Amongst XP patients, XPC patients have a lost or mutated XPC protein, the main initiator of GG-NER. Not only do they suffer from cancer in photo-exposed areas, but also XP-C patients are characterized by a 10 to 20-fold increased risk of developing internal malignancies in photo-protected sites (Zebian et al., 2019). Analysis of these internal tumors indicated that mutations are most likely caused by unrepaired oxidative DNA damages (Melis et al., 2013b). Remarkably, primary internal tumors (such as lung, uterus, thyroid, breast, and thyroid malignancies) have also been reported in XP-C patients (Hosseini et al., 2015). This predicts that XPC is involved in pathways other than NER. In this context, it has been demonstrated that NER is engaged in processing oxidative DNA lesions that are usually repaired by the BER pathway (Hutsell and Sancar, 2005). It is suggested that this role could be done by XPC (Murray et al., 2016). Researchers had started to suggest a direct link between XPC, BER, and oxidative DNA damage. For instance, it has been reported that XPC mutation leads to 8-oxoguanine (8-oxoGua) persistence, where this effect can be inverted by XPC-overexpression (D’Errico et al., 2006). Moreover, it has been demonstrated that XPC knockdown in normal keratinocytes leads to metabolism alterations through NADPH-oxidase-1 (NOX1) and ROS upregulation (Rezvani et al., 2011). Furthermore, a previous study proposed that XPC recognizes oxidative DNA damage directly, and thus it will be recruited solely without other GG-NER factors (Hosseini et al., 2015). Others demonstrated that XPC stimulates the activities of distinct glycosylases (such as OGG1 and MPG) (Zebian et al., 2019). This may explain the different cancer etiology in patients where increased intracellular oxidative DNA damage may function synergistically with altered DNA repair response to promote tumorigenesis and/or premature aging (one of the major XP-C disorder’s clinical features) (Hosseini et al., 2014, 2015).

In this study, we deciphered, post-UVB-irradiation, the effect of three different XPC mutations on the expression status and activity levels of different components of BER pathway. This unraveled the adaptation of BER components to XPC mutations and could enable: (1) better understanding of skin and internal cancers’ etiology; (2) identification of risk factors in XP-C patients; and (3) provide better insights toward designing novel therapeutic or preventive strategies.

## Materials and Methods

### Primary Fibroblasts Isolation and Culture

Three unrelated patients clinically classified as classical XP-C with no associated neurologic or extracutaneous findings were included in this study (Supplementary Table S1). After the concerned Ethical Committee agreed to perform the analysis and the patients’ parents gave their informed consent, XP-C fibroblasts were isolated from punch biopsies obtained from non-exposed patients’ body sites followed by their sequencing as previously described (Soufir et al., 2010). These cells are compatible with our aim in explaining the reason for cancer development in photo-protected areas of XP-C patients. They were compared to normal primary fibroblasts (*n* = 3) extracted by our laboratory (SyMMES, CIBEST, CEA).

Fibroblasts were cultured in DMEM medium (DMEM, high glucose, GlutaMAX^TM^ Supplement, +Pyruvate, Thermo Fisher Scientific) with 10% SVF and 1% penicillin/streptomycin in falcon flasks (75 cm^2^) at 37°C in 5% CO2 incubator. 3000 cells/cm^2^ were seeded 7 days to reach 80% confluency.

### Short-Term Cytotoxicity Assay, MTT

3-(4,5-dimethylthiazol-2-yl)-2,5-diphenyltetrazolium bromide (MTT) assay (SIGMA) was used to evaluate cell viability 24 h post-UVB-irradiation. 200 μL MTT (5 mg/mL)/well were added to six well-plates followed by a 2 h-incubation at 37°C and discarding the supernatant. Next, 2 mL DMSO/well was added along with 20 min shaking. Solutions were then transferred to 96 well microplates and the absorbance of formazan crystals at 560 nm was measured by a spectrophotometer (Spectramax M2; from Molecular Devices) allowing us to quantify the number of living cells. All data were normalized by comparison with the yield of MTT conversion in non-irradiated control samples set at 100% viability.

### Immunofluorescence and Associated Microscopy

Cells were seeded in 96 well microplates, after that exposed to UVB-irradiation at a dose of 0.03 J/cm^2^. Following that, cells were fixed at different time points (0 and 24 h) with 4% paraformaldehyde, and then permeabilized with 0.2% Triton X-100. After washing with PBS, DNA was denatured with 2 M HCL, followed by blocking with 3% FBS in PBS. The primary anti-pyrimidine (6–4) pyrimidone photoproducts (64M-2, Cosmo Bio) and secondary antibodies (Alexa Fluor 488 goat anti-mouse, Invitrogen) were diluted in 1% FBS and incubated with three washing steps between them. Finally, nuclear DNA was counter-stained with Hoechst (Sigma-Aldrich). Cell images were acquired by the Cell-insight NXT high content screening platform at 10× magnification. Data were normalized against non-irradiated samples.

### Treatment and Cell-Pellets Preparation

Sub-confluent cells (80%) were either exposed or not to 0.05 J/cm^2^ UVB-irradiation. Fibroblasts were harvested 4 h post-irradiation [real-time quantitative PCR (qRT-PCR) assay, western blot], centrifuged, and rinsed with PBS (Invitrogen, Carlsbad, CA, United States). Pellets were rapidly frozen at −80°C until further use.

For comet assay, cells were either exposed or not to 0.05 J/cm^2^ UVB-irradiation then harvested after 0, 2, and 24 h. Cells were then centrifuged, rinsed with PBS, and dissolved in freezing buffer (pH = 7.6) to be stored at −80°C.

### Reverse Transcription and Real-Time Quantitative PCR (qRT-PCR) Analysis

Total RNA was isolated using GenElute^TM^ Mammalian Total RNA Miniprep kit (Sigma-Aldrich) and then quantified using Nanodrop 1000 from Thermo scientific to check its integrity. Another method for assessing RNA integrity was to add 5 μL of sample/well (each sample tube consisted of 1 μL RNA, 9 μL water, and 2 μL DNA gel loading dye) in agarose gel using LT4 DNA ladder. Total RNA was considered intact when two acute 28S and 18S bands were visualized.

RNA (2 μg) was reversely transcribed to cDNA (Superscript^®^ III Reverse Transcriptase, Invitrogen, Carlsbad, CA, United States) in the presence of random primers (100 ng/μL, Promega, Charbonnières, France), dNTP mix (10 mM, Sigma-Aldrich, Saint-Quentin-Fallavier, France), 5×-First-strand buffer (Invitrogen, Carlsbad, CA, United States), DTT (0.1 M, Invitrogen, Carlsbad, CA, United States), ribonuclease inhibitor (45 U/μL, Sigma-Aldrich, Saint-Quentin-Fallavier, France) and SuperScript III enzyme (200 units, Invitrogen, Carlsbad, CA, United States).

Next, 5 μL of each cDNA (25 ng/μL) was used in qPCR reactions with gene-specific primers (Supplementary Table S2), and qPCR was performed by MESA Blue qPCR MasterMix Plus for SYBR^®^ Assay with low ROX (Eurogenetic, Angers, France). Samples were run in triplicates through Bio-Rad CFX96^TM^ Real-time Sys (C1000 Touch^TM^ Thermal Cycler). At the end of each run, the integrity of amplification was verified by a single melt-curve peak per product. Expression levels of target genes were normalized to those of the housekeeping gene glyceraldehyde-3-phosphate dehydrogenase (GAPDH). Calculations for determining the relative level of gene expression were made using the ΔΔCT method for quantification as reported by Livak and Schmittgen (2001).

### Western Blot

Total proteins were extracted from fibroblasts upon adding 100 μL of lysis buffer to the cell pellet followed by vortexing and incubation on ice for 30 min (vortexed every 10 min). The mixture was then transferred to 1.5 mL eppendorf and centrifuged at 16000 rpm for 15 min at 4°C. Total proteins were dosed by microBC assay protein quantification kit according to the manufacturers’ instructions. Western blotting was performed as previously described (D’Errico et al., 2006). Briefly, equal amounts of proteins were resolved by SDS-PAGE and transferred to nitrocellulose membrane (*Trans*-Blot^®^ Turbo^TM^ Transpack, Bio-Rad), followed by blocking the membrane with 5% lyophilized milk and the addition of 1/1000 diluted primary anti-XPC (mouse monoclonal antibody; Thermo Fisher Scientific), 1/50000 diluted primary anti-OGG1 (rabbit monoclonal antibody; Abcam), 1/250 diluted primary anti-MYH (rabbit monoclonal antibody; Novus Biologicals), and 1/1000 diluted primary anti-APE1 (rabbit monoclonal antibody; Sigma Aldrich). Incubation at 4°C for overnight was done, followed by incubation with mouse or rabbit anti-HRP (1/10000 diluted secondary antibody) and the addition of clarity^TM^ western ECL substrate (Bio-Rad). The membrane was visualized through Bio-Rad Molecular Imager^®^ ChemiDoc^TM^ XRS + using Image Lab^TM^ software. After a stain-free total protein detection, target proteins’ expression was normalized to the total protein extract.

### Comet Assay ± FPG

Comet assay is a single-cell gel electrophoresis assay that is used to measure the DNA lesions of cell extracts, and consequently, can monitor the excision repair capacity when it is employed at various time points post-treatment of cells. More specifically, upon adding FormamidoPyrimidine [fapy]-DNA Glycosylase (FPG) enzyme, we were able to detect 8-oxoguanine excision activity. Hydrogen peroxide (H_2_O_2_, 400 μM) was used as an internal positive control. Cells were plated in 100 mm dishes as triplicates and irradiated with 0.05 J/cm^2^ UVB-irradiation then collected after 0, 2, and 24 h. Immediately following treatment, cells were harvested, counted, and suspended at a concentration of 200,000 cells in 100 μL freezing buffer. Samples were stored at −80°C until use.

Briefly, slides were prepared with normal agarose coating 1 day in advance. On the day of the experiment, cells were deposited on the slides with 0.6% solution of low-melting agarose followed by adding a coverslip, immersing in lysis buffer, and incubation for 1 h after which a three times wash with Tris–HCL 0.4 M (neutralizing buffer) was done. FPG 0.05 u/μL (1.25 μL/slide) was then prepared in which 100 μL FPG solution with or without FPG enzyme was deposited on the slides and covered with coverslips. The slides were set on a humidified bed and added in a 37°C incubator for 40 min. The reaction was stopped by incubation for a few minutes on ice. After digestion, the slides were transferred to an electrophoresis tank filled with electrophoresis buffer pre-chilled at 4°C. The slides were left at room temperature for 30 min, and electrophoresis was subsequently done for 30 min at 25 V and 300 mA. The slides were then rinsed 3 times with Tris–HCL 0.4 M. 50 μL of Gel Red was added per slide, and a coverslip was added for reading the next day. We read the slides using a 10× objective microscope and Comet Assay IV software (Perceptive Instruments, Suffolk, United Kingdom). 50 randomly selected nuclei were scored in each slide and triplicate slides were processed for each experimental point. The extent of damage was evaluated by the Tail DNA value defined as the percentage of DNA in the tail of the comet. The normalization was done by doing a ratio of irradiated/non-irradiated at each condition.

### Statistical Analysis

The data were expressed as mean ± SEM for three independent experiments. Statistical significance of data was assessed using the student’s-*t*-test (GraphPad Prism 8) after checking variance homogeneity with the Levene’s test and normality by normality test. Student paired-*t*-test allows us to compare each sample between two different conditions while student unpaired-*t*-test allows us to compare different samples at each condition. Results were considered significant for *p*-value ≤ 0.05.

## Results

### Characterization of Normal and XP-C Primary Fibroblasts

#### Dysregulated XPC mRNA and Protein Expression Levels in XP-C Fibroblasts Compared to Normal

**FIGURE 1:**
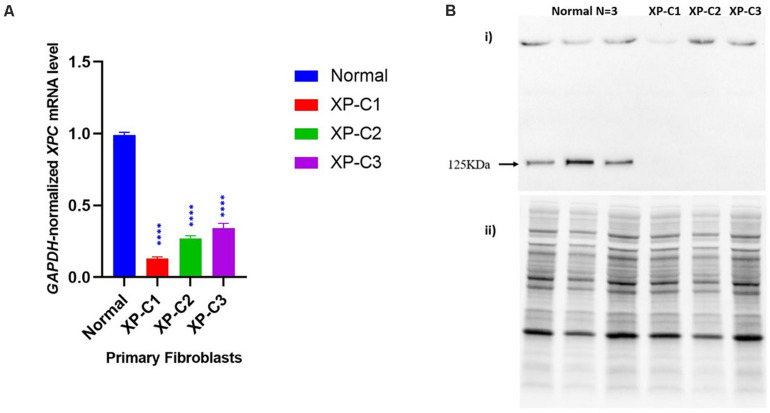
Impaired *XPC* mRNA and protein levels in XP-C primary fibroblasts at basal level. **(A)** *XPC* mRNA level is downregulated in XP-C primary fibroblasts compared to normal control at basal level. Upon qRT-PCR, all three XP-C fibroblasts exhibited a significantly reduced *XPC* gene transcription compared to the normal (*n* = 3). (*p* < 0.0001 ^****^, Unpaired-*t*-test, GraphPad Prism 8). The data were normalized relative to the GAPDH mRNA levels, where GAPDH was used as an endogenous control. The results are the mean ± SEM from three independent experiments, *n* = 3. **(B)** XPC protein was not expressed in XP-C primary fibroblasts at basal level. Although XPC protein was readily observed in all three normal fibroblasts, it was undetectable in the three XP-C fibroblasts at MW = 125 KDa. The results correspond to the mean ± SEM from three independent experiments, *n* = 3. (i) membrane with XPC bands upon hybridization with anti-XPC (ii) total protein-membrane used for normalization.

We first examined using qRT-PCR the mRNA levels of *XPC* in the normal versus XP-C fibroblasts at basal state. As shown in Figure 1A, XP-C1, XP-C2, and XP-C3 exhibited a drastically significant (*p* < 0.0001) lower *XPC* mRNA levels than normal fibroblasts (*n* = 3) (∼ 8, 4, and 3-fold downregulation, respectively). In a next step, western blot was performed to validate this expression profile at protein level. As seen in Figure 1B, XPC protein (band size = 125 KDa) was detected in normal cells but was totally absent in the three XP-C fibroblasts. This indicates that all the three different XPC mutations led to an impairment in XPC gene expression and absence at the protein level.

#### Similar Photosensitivity Between Normal and XP-C Primary Fibroblasts

**FIGURE 2:**
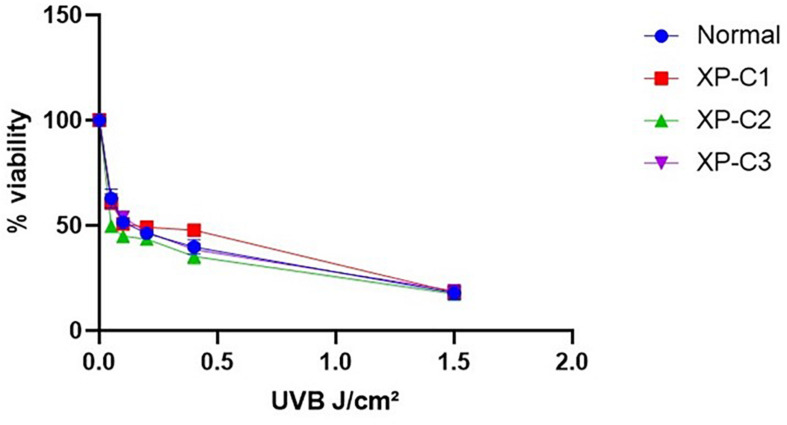
Similar Photosensitivity between normal and XP-C primary fibroblasts. Short-term cytotoxicity test (MTT) was done 24 h post-UVB-irradiation. This was done by comparing the cellular viability between normal and XP-C fibroblasts at each UVB dose condition. Each sample was normalized by its non-irradiated value (100% viability). Unpaired-*t*-test was used to compare photosensitivity between normal and each XP-C fibroblast at each UVB dose (GraphPad Prism 8). The results are the mean ± SEM from three independent experiments, *n* = 3.

We checked whether XP-C and normal fibroblasts differ in their photosensitivity as the former are suspected to be hyper-photosensitive. Hence, we did a cytotoxicity test 24 h post-UVB-irradiation. The viability of the different cells was gradually decreasing in a manner dependent on the increasing UVB doses. Generally, there was no significant difference in photosensitivity between the control and XP-C fibroblasts (Figure 2). At the highest dose (1.5 J/cm^2^), less than 20% of cells survived.

The LD_50_ was determined for all primary fibroblasts (Supplementary Table S3) using regression analysis.

Based on this cytotoxicity test, we decided to perform our experiments at 0.05 J/cm^2^ UVB dose. It is a moderate cytotoxic dose which kills <50% of cells and is thus suitable for the investigation of DNA oxidative lesions and their repair.

#### Dysregulated Photoproducts’ Repair in XP-C Primary Fibroblasts Compared to Normal

**FIGURE 3:**
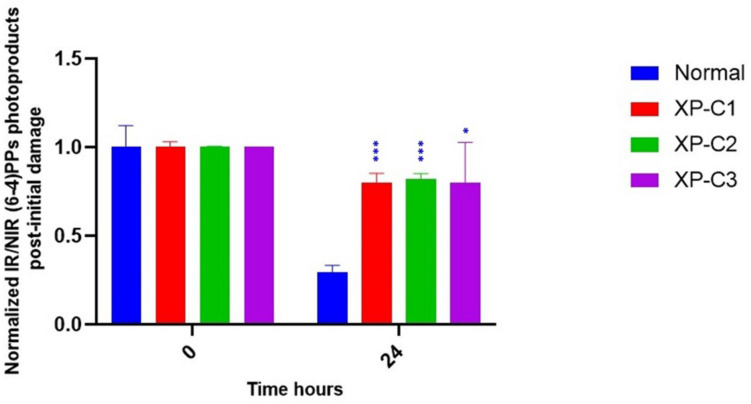
Downregulated repair of (6–4) PPs, bulky photoproducts, in XP-C fibroblasts compared to normal control. Immunocytochemistry was done to detect (6–4) PPs by fixation instantaneously at 0 and 24 h post-UVB-irradiation (0.03 J/cm^2^). An absence of primary antibody was used as negative control. The nuclei were stained with Hoechst and the (6–4) PPs were detected by green fluorescently labeled primary antibody. Images were shown upon merging both fluorescence, thereby, lesions were quantified (fluorescence signal) and normalized by non-irradiated conditions. XP-C1, XP-C2, and XP-C3 showed a significant persistence of lesion repair at 24 h compared to normal (*p* < 0.001,^∗∗∗^; *p* < 0.001,^∗∗^; *p* < 0.05,^∗^ respectively). Unpaired-*t*-test was used to compare normalized IR/NIR lesion between normal and each XP-C fibroblast at each UVB dose (0 and 24 h) (GraphPad Prism 8). The results are the mean ± SEM from two independent experiments, *n* = 2 (each experiment is done as a triplicate). IR, irradiated, NIR, non-irradiated.

Xeroderma Pigmentosum C protein does not recognize the lesion itself but rather binds to the associated helix distortion. Hence, XPC binds with a high affinity to pyrimidine (6–4) pyrimidone photoproducts [(6–4) PPs], inducing high helical alterations (Nemzow et al., 2015). For that, we were interested in following the kinetics of repair of (6–4) PPs by immunocytochemistry, where an anti-(6–4) PP was used to detect (6–4) PPs at 0 and 24 h. 24 h post-UVB-irradiation around 70% of lesions were repaired in normal fibroblasts. However, this was not the case in the three XP-C fibroblasts where elevated levels persisted. Almost 20% were repaired in XP-C1, XP-C2, and XP-C3 as shown in Figure 3 and Supplementary Figure S1. This lesion persistence was significant in XP-C1, XP-C2, and XP-C3 compared to normal fibroblasts (*p* < 0.001, *p* < 0.001, *p* < 0.05, respectively).

### Dysregulated BER-Associated Gene Expression in XP-C Fibroblasts Compared to Normal, Post-UVB-Irradiation

**FIGURE 4:**
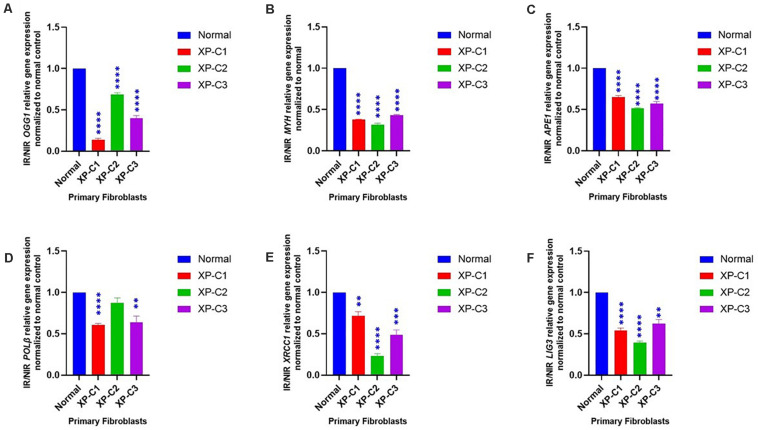
Downregulated BER-associated gene transcription in normal and XP-C fibroblasts, post-UVB-irradiation. Gene transcription was investigated by qRT-PCR experiments in XP-C vs. control fibroblasts 4 h post-UVB dose (0.05 J/cm^2^). Total RNA extraction was followed by reverse transcription. QRT-PCR was carried out to assess gene expression. Shown values are the mean ± SEM from three independent experiments, *n* = 3. The used calibrator was non-irradiated normal fibroblast where expression ratios were normalized by that of control. Ratio of IR/NIR was used in analysis. Panel **(A)** shows the significant downregulation of normalized IR/NIR *OGG1* gene expression in XP-C fibroblasts compared to normal (*p* < 0.0001,^****^). Panel **(B)** shows the significant downregulation of normalized IR/NIR *MYH* gene expression in XP-C fibroblasts compared to normal (*p* < 0.0001,^****^). Panel **(C)** shows the significant downregulation of normalized IR/NIR *APE1* gene expression in XP-C fibroblasts compared to normal (*p* < 0.0001,^****^). Panel **(D)** XP-C1 and XP-C3 showed a significant *Pol*β downregulation compared to normal (*p* < 0.0001,^****^ and *p* < 0.01,^∗∗^ respectively) while no significant difference was observed while comparing XP-C2 to the control. Panel **(E)** shows the significant downregulation of normalized IR/NIR *XRCC1* gene expression in XP-C1, XP-C2, and XP-C3 compared to normal (*p* < 0.01,^∗∗^; *p* < 0.0001,^****^ and *p* < 0.001,^∗∗∗^ respectively). Panel **(F)** shows the significant downregulation of normalized IR/NIR *LIG3* gene expression in XP-C1, XP-C2, and XP-C3 compared to normal (*p* < 0.0001,^****^; *p* < 0.0001,^****^ and *p* < 0.01,^∗∗^ respectively). This was done by unpaired-*t*-test that allows the comparison between normal and each XP-C fibroblast (GraphPad Prism 8). IR, irradiated; NIR, Non-Irradiated.

We examined the mRNA levels of a series of genes involved in BER between normal and XP-C primary fibroblasts 4 h post-UVB-irradiation (Figure 4). We scanned the whole BER pathway starting from the initiation and base removal steps (*OGG1*, *MYH*), passing by abasic sites removal (*APE1*), and to newly synthesized nucleotide (*PolB*) and ligation (*LIG3*, *XRCC1*).

We observed that 8-oxoguanine glycosylase (*OGG1*), MutY Adenine DNA Glycosylase (*MYH*), apurinic endonuclease 1 (*APE1*), ligase 3 (*LIG3*), and X-ray repair cross-complementing 1, LIG3’s cofactor, (*XRCC1*) were characterized by a significantly lower mRNA levels in all three XP-C fibroblasts compared to normal cells (*p* < 0.01). On the other hand, *PolB* transcription levels were significantly downregulated in XP-C1 and XP-C3 (*p* < 0.01) but not XP-C2.

### Dysregulated BER-Associated Protein Expression in XP-C Fibroblasts Compared to Normal, Post-UVB-Irradiation

**FIGURE 5:**
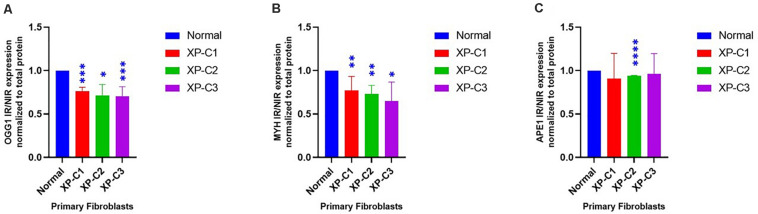
Downregulated BER-associated protein levels in normal and XP-C fibroblasts, post-UVB-irradiation. Protein level was investigated in XP-C vs. control fibroblasts 4 h post-UVB-irradiation (0.05 J/cm^2^). Total protein was extracted followed by western blot to evaluate protein expression. Values shown are the mean ± SEM from three independent experiments, *n* = 3. Ratio of IR/NIR was used in analysis after normalization by the total protein. Panel **(A)** shows the significant downregulation of normalized IR/NIR OGG1 protein expression in XP-C1, XP-C2, and XP-C3 compared to normal (*p* < 0.001,^∗∗∗^; *p* < 0.05,^∗^; and *p* < 0.001,^∗∗∗^). Panel **(B)** shows the significant downregulation of normalized IR/NIR MYH protein expression in XP-C1, XP-C2, and XP-C3 compared to normal (*p* < 0.01,^∗∗^; *p* < 0.01,^∗∗^ and *p* < 0.05,^∗^ respectively). Panel **(C)** shows the significant downregulation of normalized IR/NIR *APE1* gene expression in XP-C2 fibroblast compared to normal (*p* < 0.0001,^****^). Statistical analysis was done by unpaired-*t*-test that allows the comparison between normal and each XP-C fibroblast (GraphPad Prism 8). IR, irradiated; NIR, Non-Irradiated.

To better understand the effect of XPC mutation on BER’s regulation, we studied the difference in OGG1, MYH, and APE1 protein levels between normal and XP-C primary fibroblasts 4 h post-UVB-irradiation (Figure 5 and Supplementary Figure S2). Such proteins were selected due to their main role in initiating BER of oxidative damage. OGG1 and MYH were characterized by a significantly (*p* < 0.05) lower protein levels in all three XP-C fibroblasts compared to normal cells. Meanwhile, APE1 protein expression was significantly downregulated in XP-C2 (*p* < 0.0001) but not XP-C1 and XP-C3.

### Lower Intrinsic Base Excision-Repair Capacities in XP-C Primary Fibroblasts Compared to Normal

**FIGURE 6:**
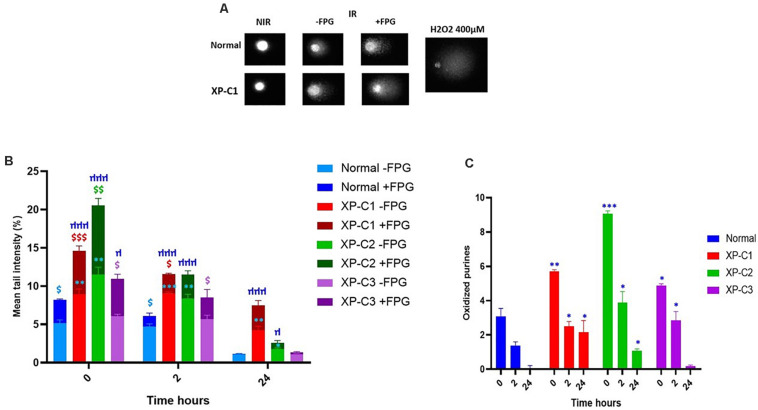
Low intrinsic base excision excision-repair capacities in XP-C primary fibroblasts. Comet ± FPG was done to detect single-strand breaks (SSB), alkali-labile sites (ALS), and oxidative purines (including 8-oxoGua) in each sample at each condition. **(A)** Illustrates the undamaged (comet head) and damaged (comet tail) DNA ± FPG in normal and XP-C1 fibroblasts and positive control H_2_O_2_; the% tail intensity and length are proportional to the DNA damage. **(B)** The graphical representation displays the mean tail intensities (%) for each sample, for both FPG active sites (dark-colored) and SSB/ALS (light-colored) post-UVB-irradiation (0.05 J/cm^2^). All fibroblasts were able to repair; however, the XP-C fibroblasts had a downregulated and dwindled repair activity. We did a ratio of IR/NIR ± FPG for each fibroblast at three kinetic points = 0, 2, and 24 h. The results are the mean ± SEM from three independent experiments. Paired-*t*-test was done to compare each sample with two conditions (FPG–ve or FPG+ve). Unpaired-*t*-test was done to compare different samples within the same condition (GraphPad Prism 8). $ Sample significantly (*p* < 0.05) higher in its tail intensity with presence of FPG (+FPG) compared to its absence (–FPG). ^∗^XP-C fibroblast significantly (*p* < 0.05) higher in its tail intensity compared to normal fibroblast, at –FPG condition. μ XP-C fibroblast significantly (*p* < 0.05) higher in its tail intensity compared to normal fibroblast, at +FPG condition. **(C)** The graphical representation displays oxidized purines repair (8-oxoGua and Fapy) in normal compared to XP-C fibroblasts. These oxidized purines were detected upon subtracting values +FPG from values –FPG for each sample. As expected, oxidized purines were downregulated in all fibroblasts. At *t* = 0 h, XP-C1, XP-C2, and XP-C3 had higher oxidized purines compared to normal fibroblasts (*p* < 0.01,^∗∗^; *p* < 0.001,^∗∗∗^ and *p* < 0.05,^∗∗^ respectively). Similarly was shown at *t* = 2 and 24 h for XP-C1, XP-C2, and XP-C3; except for XP-C3 at 24 h. Shown values correspond to the mean ± SEM from three independent experiments. Unpaired-*t*-test was done to compare different samples within the same condition (GraphPad Prism 8). ^∗^XP-C fibroblast significantly (*p* < 0.05) higher in its oxidized purines compared to normal fibroblast. –FPG = FPG alkaline buffer without the enzyme. +FPG = FPG alkaline buffer and FPG enzyme. IR, irradiated, NIR, Non-Irradiated.

Standard alkaline comet (−FPG) is a genotoxic assay that measures DNA single-strand breaks (SSBs) and alkali-labile sites (ALS). Once FPG glycosylase is added, oxidized purines (including 8-oxoguanine) can be evaluated. This is done by the excision of FPG-sensitive sites (oxidized purines) converting abasic sites into DNA SSBs. The net cleavage sites (oxidized purines) generated by FPG activity are calculated by subtracting the value of DNA damage at alkaline conditions from that with FPG treatment (as presented in Figure 6C). This FPG enzyme is functionally similar to OGG1 where both recognize oxidized purines, majorly Fapy and 8-oxoGua (Hu et al., 2005).

Figure 6A shows an example of comets done ± FPG in normal and XP-C1 fibroblasts, and the positive control, H_2_O_2_. The head of comet represents intact DNA, meanwhile, the tail represents damaged DNA.

#### In Absence of FPG

Ultraviolet B rays-irradiation increased SSBs to the maximum at time = 0 h in all primary fibroblasts. However, this was significantly higher in XP-C1 and XP-C2 compared to the control (*p* < 0.01, ^∗∗^). On the contrary, the increase of DNA damages in XP-C3 was not significant compared to the control. During the course of the experiment, lesions were repaired faster in the normal fibroblasts. Similarly, at times = 2 and 24 h, XP-C1 and XP-C2 had significantly higher DNA lesions compared to normal (*p* < 0.01 and *p* < 0.05, respectively) (Figure 6B).

#### In Presence of FPG

Upon adding FPG,% mean tail intensity increased due to more breaks in DNA where FPG will specifically excise oxidized purines (oxidative DNA lesions, as 8-oxoguanine and Fapy). This was clearly demonstrated upon a significant increase in intensity when comparing the samples with/without FPG at times = 0 and 2 h (*p* < 0.05, $). When comparing control cells to each XP-C fibroblast: significantly higher DNA lesions were observed in XP-C1 and XP-C2 at *t* = 0, 2 and 24 h. Meanwhile, XP-C3’s higher DNA lesions were only significant at *t* = 0 h (*p* < 0.05, μ) (Figure 6B).

Figure 6C is a zoom in to Figure 6B. It represents oxidized purines present in each sample and its kinetic repair follow up. At *t* = 0 h, XP-C1, XP-C2, and XP-C3 had higher oxidized purines compared to normal control (*p* < 0.01, *p* < 0.001, and *p* < 0.05, respectively). Similarly, at *t* = 2 and 24 h, XP-C fibroblasts showed significantly higher oxidized purines compared to control (*p* < 0.05); except for XP-C3 at 24 h.

On the contrary to Berra et al. (2013), the repair was not similar between XP-C deficient and XP-C-proficient fibroblasts (normal control). Induction of single strand breaks and oxidized purines was more prominent and persistent (slower rate of repair) in XP-C fibroblasts compared to control at 0, 2, and 24 h where both lesion types are usually repaired by BER.

## Discussion

It is well described that several cancer-prone diseases result from defective nucleotide excision DNA repair. This is well known for XPC mutations that are associated with high rate of basal cell carcinomas (BCCs), squamous cell carcinomas (SCCs), and melanoma in photo-exposed skin (De Boer and Hoeijmakers, 2000).

In a previous report, Agar et al. was able to detect 8-oxoGua and its correlated G:C→T:A transversion, thus indicating the contribution of such oxidative DNA damage to skin cancer (D’Errico et al., 2006). Hence, they speculated that the increased UV-induced skin cancer could be attributed not only to reduced NER but also to impaired BER, the foremost oxidative DNA damage repair system (D’Errico et al., 2006). Besides, XP patients also suffer from internal cancers that could be contributed to ROS accumulation and oxidative stress. Indeed, exploiting the long term follow-up of XP patients at NIH (National Institute of Health) from 1971 to 2009, Bradford et al. (2010) have reported that internal cancers (17%, *n* = 5) could be considered as the third factor [besides skin cancer (34%, *n* = 10) and neurologic degeneration (31%, *n* = 9)] leading to XP patients’ death. Of note, among XP patients in their cohort, only patients belonging to XP-C group (6 out of 12) died due to internal cancers, including central nervous system cancers (*n* = 3), peripheral nerve cancer (*n* = 1), lung cancer (*n* = 1) and endocervical adenocarcinoma of the uterus (*n* = 1). Furthermore, Live imaging data has shown that XPC is rapidly recruited to the oxidized bases, independent on the recruitment of downstream NER factors (Menoni et al., 2012).

Thus, our study aimed to assess the effect of different XPC-mutations on BER in order to decipher how its phenotype is linked to a defect in BER’s DNA repair capacity.

### Characterization of Normal and XP-C Primary Fibroblasts

In this study, we used fibroblasts isolated from patients with different clinical manifestations. XP-C1 had frame-shift mutation (c.1643_1644del). Meanwhile, XP-C2 and XP-C3 patients were compound heterozygotes for *XPC* mutations (Supplementary Table S1). As shown in Figure 1, while the full-length XPC protein was undetectable in fibroblasts from the three XP-C patients, XPC mRNA was expressed in these cells although at a significantly lower level than that of control fibroblasts. This suggests that XPC-mutated mRNAs may contain premature termination codons (PTCs) that induce non-sense mediated mRNA decay (NMD pathway) as a protective method to prevent deleterious-truncated proteins’ expression. Our observations are in agreement with what was described by Chavanne et al. (2000); Khan et al. (2005), and Senhaji et al. (2012) who reported that mutations in the *XPC* gene are expected to cause protein truncations as a result of non-sense, frameshift, and deletion events. Thus, XPC mRNA levels may be considered as a predictive-diagnostic biological marker protecting from skin cancer since its low expression level is linked to an increased susceptibility to cancer in XPC-mutation carriers (Khan et al., 2005).

Even though photosensitivity is a XP-linked symptom, it was not the case when we did the short-term cytotoxicity test on XP-C fibroblasts. Similarly to De Waard et al. (2008), all fibroblasts shared similar moderate UVB-photosensitivity. It was clearly demonstrated that *XPC* mutations and deletions do not shorten lifespan in mice rather perhaps could be early events that induce late onset, slow growth/progression of tumor (Hollander et al., 2005). This suggests that cells harboring accumulated UVB-induced DNA damage are not eliminated with apoptosis. In agreement, Hollander et al. (2005) had shown that *Xpc*^–/–^ mice develop spontaneous lung tumors at old age, due to an overtime accumulating effect passing the threshold of cancer risks. Also, Rezvani et al. (2006) showed that XP-C cells underwent spontaneous tumoral transformation owing to their susceptibility to accumulate DNA damage. XPC protein is not essential for cellular viability, proliferation or development as its dysfunction does not result in stalling RNA replication fork; hence, the difference between normal and mutated fibroblasts is the persistence of mutations in the latter arising to genomic instability and abnormal survival. In accordance, XP-C patients have been reported to suffer less from acute burning on minimal sun exposure than other XP complementary groups (Chavanne et al., 2000; Sethi et al., 2013). Indeed, it has been suggested that XP-C patients are diagnosed later in their life owing to their normal sunburn reactivity compared to the other XP groups. Consequently, XP-C patients are less likely to adhere to ultra-violet radiation protection and precaution early in their lives. As the inevitable consequence, they later develop a more aggressive phenotype compared to other XP-groups (Fassihi et al., 2016). This implies that sensitivity to sunburn may not always be an adequate clinical marker of an individual’s skin cancer risk rather NER capacity, GG-NER in particular, may be a better predictor (Berg et al., 1998).

Hence, we monitored the repair of bulky lesions in normal and XP-C fibroblasts to investigate XPC mutations’ effect on GG-NER repair activity. XPC protein’s binding affinity to DNA correlates with the extent of helical distortions. Although it recognizes (6–4) PPs and CPDs, it binds with more specificity to the former that are bulkier than CPDs (Melis et al., 2008). Thus, we decided to study the effect of XPC mutations on the repair of (6–4) PPs at 0 and 24 h post-UVB-irradiation. Repair synthesis in XPC-mutated cells ranges between 10 and 20% compared to normal (Chavanne et al., 2000). In agreement with that, we showed that only ∼20% of pyrimidine (6–4) pyrimidone photoproducts’ [(6–4) PPs] were repaired in XP-C fibroblasts after 24 h. This is consistent with what Courdavault et al. (2004) had published. (6–4) PPs were repaired efficiently within less than 24 h in cultured dermal human primary fibroblasts.

### Downregulation of Different BER-Associated Gene and Protein Levels in XP-C Fibroblasts Compared to Normal

Besides skin cancers, XP patients have a 10 to 20-fold increased risk of developing internal malignancies, such as lung, tongue, brain, and liver cancer (Bowden, 2004; DiGiovanna and Kraemer, 2012; Zhang et al., 2015; Murray et al., 2016; Zebian et al., 2019). These incidences cannot be explained unless XPC acts as a multi-functional protein involved in roles beyond GG-NER initiation. It recognizes both NER targeted lesions and base lesions which may provide it the power of determining the eventual repair type. Once cells are irradiated by UVB, stress-mediated alterations in mitochondrial and nuclear functions and oxidative unbalance will arise since almost 50% of UVB-induced lesions attribute to the formation of ROS, consequently oxidative DNA damage and cancer. These events are highly pronounced once XPC protein is dysregulated (Rezvani et al., 2011; Wölfle et al., 2011; Melis et al., 2013a; Hosseini et al., 2014; Zhang et al., 2015). For instance, there is an association between the increased lung tumor incidence and oxidative stress in *Xpc*-knock out mice (Liu et al., 2010). D’Errico et al. (2006) showed that primary keratinocytes and fibroblasts derived from XP-C patients are hypersensitive to DNA-oxidizing agents and that could be inverted by the re-expression of XPC. In good agreement, the activity of catalase, an enzyme protecting the cell from oxidative DNA damage through the conversion of H_2_O_2_ into oxygen and water, was found to be decreased in XP patients (Rezvani et al., 2006). XPC has been reported to affect oxidative and energy metabolism. For instance, *in vitro* studies displayed an elevated sensitivity in *Xpc*^–/–^ mouse embryonic fibroblasts (MEFs) to oxidative DNA-damaging agents compared to control (Melis et al., 2008). It is estimated that up to 100,000 8-oxoGua lesions can be formed daily in DNA per cell (Ba and Boldogh, 2018). They are recognized by OGG1 and MYH. The former excise 8-oxoGua directly, meanwhile, the latter removes misincorporated adenines in front of 8-oxoguanine (8-oxoGua) during DNA replication. These excision activities will result in an AP site that will be cleaved by APE1 for the synthesis and ligation to be carried out by POLβ and LIG3, respectively (Campalans et al., 2015). It has been shown that MYH-OGG1 deficient cells are sensitive to oxidants and ROS (Xie et al., 2008). As ROS accumulation negatively regulates the activity of several important DNA repair proteins, including OGG1, its production may lead to the increase of DNA damage which supports the important role of ROS in carcinogenesis. Not only it induces oxidative DNA damage but also prevents its repair (Srinivas et al., 2018). Mutations in the *OGG1* gene can lead to lung and kidney tumors and the S326C polymorphism appears to be associated with an increased risk of esophageal, lung and prostate cancers (Thibodeau et al., 2019). In parallel, mutations in the *MYH* gene are associated with lung, colorectal and breast cancers (Hollander et al., 2005; Viel et al., 2017; Thibodeau et al., 2019). In light of these studies, several questions arise. Would this occur through lack of XPC-BER interaction? If yes, how profound is XPC’s influence on BER effectiveness and expression?

Therefore, we were interested in highlighting the role of XPC as an interplay between NER and BER. For that, real-time-qPCR was done where primers anneal to each of the following BER components: *OGG1*, *MYH*, *APE1*, *POL*β, *XRCC1*, and *LIG3*. Afterward, we did western blot analysis on OGG1, MYH, and APE1. These proteins were selected due to their essential role in initiating oxidative DNA lesions’ repair.

OGG1, MYH gene and protein expressions were significantly inhibited upon UVB-irradiation (*p* < 0.05). Although APE1 showed a significantly inhibited gene expression in all XP-C fibroblasts (*p* < 0.0001), such an inhibition was not significant at protein level except in XP-C2. These results indicate a downregulation in stimulation. In agreement with our results, it has been shown that XPC is recruited to 8-oxoguanine lesions to induce a partial removal of the oxidative DNA damage and regulate cellular stress-response (Zhou et al., 2001; Miccoli et al., 2007). This is done by stimulating OGG1’s protein expression and catalytic activity and physically interacting with APE1 (D’Errico et al., 2006; De Melo et al., 2016). Hence, XPC mutation affects them directly. For example, there is an evidence that *XPC* P334H substitution can prevent stimulation of BER factor OGG1 (Melis et al., 2013b). However, little is mentioned in the literature about the effect of XPC on *MYH*, *LIG3*, *POLB*, and *XRCC1*. Perhaps MYH was barely studied because it is an indirect secondary actor in the repair of 8-oxoGua, functioning downstream of OGG1 and removing adenine bases misincorporated opposite 8-oxoGua (Forestier et al., 2012). However, due to its role, there might be a direct link between MYH and XPC. This was seen at both gene and protein levels. Meanwhile, other BER factors’ mRNA downregulation could be explained by the fact that an interaction and cross-talks amongst BER factors is crucial for the recruitment to the site of repair and optimum repair efficiency (Campalans et al., 2015). Hence, since, as shown in our results, OGG1, MYH, and APE1 are affected, a stimulation to trigger the expression of downstream factors could be inhibited or slowed down where the coordination amongst the protein complexes is similar to passing of the baton, where the repair product is handed over from an enzyme to the next one.

### Downregulation in Excision Activity of BER-Associated Enzymes in XP-C Fibroblasts Compared to Normal

Some studies have demonstrated that ROS-induced 8-oxoguanine formation, primarily in guanine-rich gene regulatory regions, inactivates OGG1’s enzymatic activity (Hao et al., 2018), resulting in GC to TA transversion mutations (De Rosa et al., 2012). Hence, 8-oxoGua accumulation might be considered as a diagnostic marker for BER malfunction (Tinaburri et al., 2018). For example, OGG1-Cys enzymatic activity decreases under oxidative stress due to redox-sensitive residues in accordance with our results where there is a reverse correlation between OGG1 activity and oxidative stress (Bravard et al., 2009; De Rosa et al., 2012; Tinaburri et al., 2018). Moreover, D’Errico et al. (2006) had shown that XPC plays a role as a cofactor for the efficient 8-oxoGua excision by OGG1. XPC/P334H mutation weakens the interaction between OGG1 and XPC, resulting in a decreased glycosylase activity and turn-over (Melis et al., 2013b). Additionally, studies had demonstrated that APE1 and XRCC1 are involved in the repair of SSBs containing 3′-8-oxoGua and SSBs, respectively in human cell extracts (Okano et al., 2000; Parsons et al., 2005). Both were shown, in our data, to be downregulated at mRNA level in absence of XPC. Decreased expression of several BER factors in XP-C cells could explain why at time = 0 h, more single strand breaks and oxidative DNA damage were found in these cells compared to control. Our results also showed that repair of the oxidative damage was much lower and slower in XP-C cells than normal cells. In agreement, it had been shown that XPC deficiency impairs the repair of oxidative DNA damage induced by visible light and methylene blue where XPC had been proven to bind much better oxidative base damage than direct SSBs (Menoni et al., 2012; Melis et al., 2013b). Similarly, the level of 8-oxoGua in cells treated with KBrO3 (40 mM) at different time points after exposure was much higher in XP-C cells compared to their control counterparts (D’Errico et al., 2006). Despite the dysregulation in BER’s efficiency in XP-C fibroblasts, a bashful repair occurred. This could be explained by two complement scenarios: Although OGG1 is the main preferred actor in BER, other multiple of backup-glycosylases will step up once it is function becomes incompetent (Hegde et al., 2008). On the other hand, as mentioned before, XPC enhances OGG1’s turnover i.e., efficiency of activity (De Melo et al., 2016). In the absence of XPC, OGG1 is stable and able to remove oxidized lesions with a less competency and slower rate.

These results suggest that increased susceptibility to internal tumors in XP-C patients and spontaneous tumors in *Xpc* mice may be due to incompetent oxidative DNA lesions repair.

It is evident now that repair of both endogenous and induced oxidative DNA damage are essential for maintaining genomic integrity and homeostasis. This involves complex interactions among BER proteins and between them and other proteins, mainly XPC (Hegde et al., 2008).

## Conclusion

The difference in XPC mutations among our samples allowed us to have a general and more confirmed conclusion about the effect of such protein on the expression and activity status of distinct BER system components to repair oxidized DNA damage.

Characterization of the interplay between BER factors and XPC may provide new insights about the occurrence of non-skin cancer upon XPC-deficiency. Furthermore, the synergic effects of amassed oxidative DNA damage and impaired BER could explain heterogeneity in the clinical spectrum of XP-C patients.

## Data Availability Statement

All datasets generated for this study are included in the article/Supplementary Material. For further inquiries contact the corresponding author.

## Author Contributions

NF performed all the experiments (cell culture, short-term cytotoxicity assay, qRT-PCR, western blot, Immunocytochemistry, and comet ± FPG assay) and wrote the manuscript. WR conceived the project, grant funding, supervised the project, and revised the manuscript. HRR contributed to formal analysis and manuscript editing and revision. MF-K and BB aided in the manuscript revision. HF-K participated in the statistical analysis and manuscript revision. FK assisted in the immunocytochemistry experiment. DB assisted in certain experiments. WM prepared the XP-C fibroblasts. They were sequenced and had their mutation identified by CG and FM-P. All authors read and approved the final version of the manuscript.

## Conflict of Interest

The authors declare that the research was conducted in the absence of any commercial or financial relationships that could be construed as a potential conflict of interest.
