# Supplementary material for: Xeroderma Pigmentosum C (XPC) Mutations in Primary Fibroblasts Impair Base Excision Repair Pathway and Increase Oxidative DNA Damage
Source: Front Genet. 2020 Nov 27;11:561687. doi: 10.3389/fgene.2020.561687 (PMC7728722; doi:10.3389/fgene.2020.561687)
Supplement: Supplementary file 1 [file Data_Sheet_1.docx]

***SUPPLEMENTARY DATA***

**Supplementary table 1. Main characteristics of the XP-C patients involved in the study**

The three patients had regular follow-up after initial diagnosis at the Dermatology Department, Pediatric Unit, Bordeaux Hospital (Reference Center for Rare diseases of the Skin) for careful examination of pre-cancerous lesions. Genotyping was performed on whole blood at the first visit, after obtaining informed consent from the parents. *XPC* gene analysis was performed by Sanger sequencing as previously described [60], and bi-allelism was confirmed by parental testing. Five different *XPC* gene defects were identified, each resulting in a premature stop, due to either a small deletion (*XP-C1*-homozygous *XPC* prevalent mutation, *XP-C2*-allele 2, *XP-C3*-allele 2) or a non-sense (*XP-C3*-allele 1). Such gene defects are the common rule in XPC disease [60]. RNA analysis was performed in patient 2, in order to confirm the splicing defect (allele 1: deletion at the splice site).

| **Patient id** | **Geographic origin** | **Main clinical features** | **XPC gene defect (NM_004628.4)** | **Age at diagnosis** |
| --- | --- | --- | --- | --- |
| XP-C1 | Morocco | Early skin photosensitivity | c.1643_1644del, p. (Val548Alafs*25) homozygous | 2 y |
| XP-C2 | Portugal | Moderate skin photosensitivity - multiple lentigines | [c.413-3delC, p.?], [c.1086del, p. (Ser363Alafs*163)]  compound heterozygous | 14 y |
| XP-C3 | West Indies/Chile | Skin photosensitivity -multiple lentigines - precancerous lesions | [c.1243C>T, p. (Arg415*)], [c.2287del, p.(Leu763Cys*4)]  compound heterozygous | 6 y |

**Supplementary table 2. Reverse and forward primers used in RT-qPCR.** The primers were used at a concentration of 2 µM in a mix with MESA Blue qPCR MASTER-MIX, distilled water and 5µL of each cDNA. Amplification integrity was checked at the end of each run by melting-curve analysis.

| **Primers** | **Forward** | **Reverse** |
| --- | --- | --- |
| ***XPC*** | CCATGAGGACACACACAAGG | TCCAATGAACCACTTCACCA |
| ***OGG1*** | TGGAAGAACAGGGCGGGCTA | ATGGACATCCACGGGCACAG |
| ***MYH*** | CCAGAGAGTGGAGCAGGAAC | TTTCTGGGGAAGTTGACCAC |
| ***APE1*** | GCTGCCTGGACTCTCTCATC | GCTGTTACCAGCACAAACGA |
| ***LIG3*** | GCTCAGCAGGAGATGGTTTC | TCTAGGTCCCGTGCCATATC |
| ***XRCC1*** | CAGCCCTACAGCAAGGACTC | GCTGTGACTGGGGATGTCTT |
| ***Polβ*** | GAGAAGAACGTGAGCCAAGC | CGTATCATCCTGCCGAATCT |

**Supplementary table 3. Measurement of LD50 for normal and XP-C primary fibroblasts upon exposure to different UVB-doses.** LD50 represents the UVB dose that will reduce 50% of the in-vitro cell survival. It is calculated after short-cytotoxicity test that represents cellular viability *vs*. UVB doses. The results represent the average of three different experiments, n=3.

| **Fibroblasts** | **LD50 J/cm²** | **LD50 for all together J/cm²** |
| --- | --- | --- |
| **Normal N=3** | 0.147 | 0.1355 |
| **XP-C1** | 0.179 |  |
| **XP-C2** | 0.064 |  |
| **XP-C3** | 0.152 |  |

**
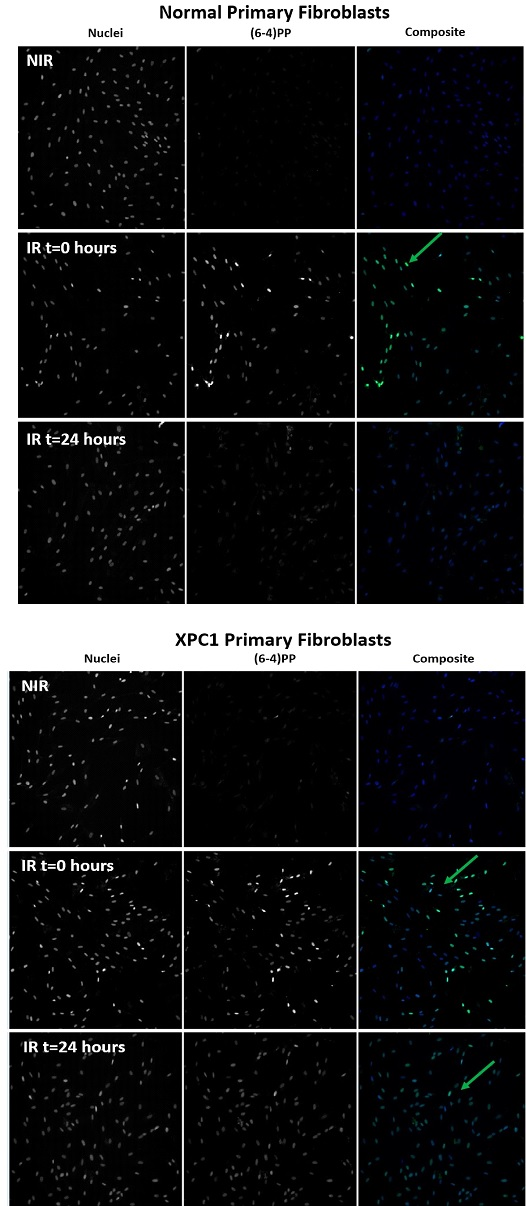
**

**Supplementary figure 1. Immunocytochemistry images showing the 6-4PP photoproducts in normal and XP-C primary fibroblast, XP-C1.** This was done at 0 and 24 hours post-UVB-irradiation where anti-(6-4) PP was used to detect how (6-4) PPs is repaired by time in control and mutated fibroblasts. Green color represents the presence of (6-4) PPs in the nuclei. Nuclei were visualized by Hoechst staining. As shown above, no lesions were detected in all fibroblasts without UVB stress. Upon UVB treatment, lesions appear at t=0 hours to be repaired almost completely in the normal fibroblasts but still present in XP-C fibroblast (XP-C1).

**
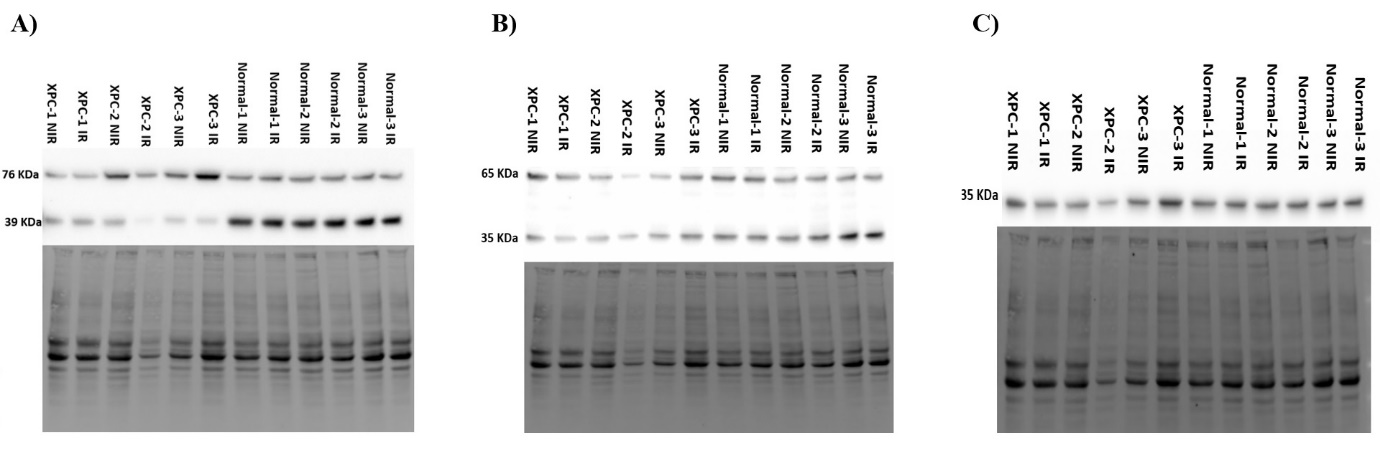
**

**Supplementary figure 2. OGG1, MYH, and APE1 protein expression in normal and XP-C primary fibroblasts at basal and UVB-irradiation levels.** A) OGG1 protein expression (76 and 39 KDa, 2 isoforms) is downregulated at basal and UVB-irradiation levels in XP-C fibroblasts compared to normal (N=3), B) MYH protein expression (65 and 35 KDa, complete and degraded protein) is downregulated at basal and UVB-irradiation levels in XP-C fibroblasts compared to normal (N=3), C) APE1 protein expression (35 KDa) is downregulated at basal and UVB-irradiation levels in XP-C2 fibroblast compared to normal (N=3). Proteins’ level intensities were detected at 4 hours post UVB-irradiation and were quantified using Image Lab^TM^ software. Target proteins’ expression was normalized to the total protein extract.

IR, irradiated, NIR, Non-Irradiated
